# The BLIM, the DINA, and their polytomous extensions. Rejoinder to the Commentary by Chiu, Köhn, and Ma

**DOI:** 10.1017/psy.2024.24

**Published:** 2025-01-03

**Authors:** Luca Stefanutti, Pasquale Anselmi, Debora de Chiusole, Andrea Spoto, Jurgen Heller

**Affiliations:** 1 FISPPA Department, University of Padua, Padova, Italy; 2 Department of General Psychology, University of Padua, Padova, Italy; 3 Department of Psychology, University of Tuebingen, Tuebingen, Germany

**Keywords:** basic local independence model, cognitive diagnosis models, deterministic input noisy AND-gate model, knowledge space theory, polytomous local independence model

## Abstract

The basic local independence model (BLIM) is a probabilistic model developed in knowledge space theory (KST). Recently, Stefanutti, de Chiusole, et al. (2020, *Psychometrika* 85, 684–715) proposed the polytomous local independence model (PoLIM), which is an extension of the BLIM to items with more than two response alternatives (polytomous items). In a Commentary to this paper, Chiu et al. (2023, *Psychometrika* 88, 656–671) claimed that (i) the BLIM is just a deterministic input noisy AND-gate (DINA) model where every item has a single skill and, as a consequence of this, (ii) the “PoLIM is simply a paraphrase of a DINA model in cognitive diagnosis (CD) for polytomous items” (p. 656). This rejoinder shows that such statements are invalid and totally misleading. Its aim is to clarify the nature of the relationship between the BLIM and the DINA, as well as that between the PoLIM and the Polytomous DINA. It builds upon formal results by Heller, et al. (2015, *Psychometrika* 80(4), 995–1019) on the intimate relation between KST and CD notions, and shows that the BLIM/PoLIM may be conceived as marginal models for whole classes of CD models.

## 1 Introduction

The basic local independence model (BLIM) is a probabilistic model developed in knowledge space theory (KST) by Falmagne and Doignon (1988). It is a restricted latent class model aimed at modeling the responses of individuals to dichotomous (correct/incorrect; true/false) items. In KST, the latent classes are called *knowledge states*. Each knowledge state is a subset *K* of a given set *P* of items, and represents all the items in *P* that an individual masters. The collection 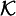 of all the knowledge states is the *knowledge structure*. In any practical application of KST, due to assumed dependencies among the items, not every subset of *P* is a knowledge state, and 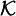 turns out to be a strict subset of the whole power set 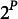 on *P*.

The observable *response pattern* of an individual to the items in *P* is represented by the subset *R* of *P* of all those items that received a correct response. The knowledge state *K* and the response pattern *R* of the same individual need not be identical, due to random error. Some items can be in *R* but not in *K* (as *false positives*) and some other items can be in *K* but not in *R* (as *false negatives*). In the BLIM, the probability 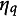 of a false positive for a given item 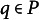 is interpreted as *lucky guess*, whereas the probability 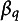 of a false negative for *P* is interpreted as a *careless error*. The most important assumption of the BLIM is that the responses of an individual to the items are locally independent, given her or his knowledge state.

Recently, Stefanutti, de Chiusole, et al. (2020) extended the BLIM to items with more than two response alternatives (polytomous items). Such an extension, called polytomous local independence model (PoLIM), can be regarded as a rather natural consequence of recent generalizations of KST to polytomous items (see, e.g., Heller, 2021; Stefanutti, Anselmi, et al. 2020). Models for polytomous items exist also in the area of cognitive diagnosis (CD), but none of those published previous to the Stefanutti, de Chiusole, et al. (2020) paper corresponds to the PoLIM.

In a Commentary to Stefanutti, de Chiusole, et al. (2020), Chiu et al. (2023) claimed that the “PoLIM is simply a paraphrase of a DINA model in cognitive diagnosis for polytomous items” (p. 656). The DINA (Deterministic Input Noisy AND-gate; Haertel, 1984, 1989, 1990) model is one of the most prominent and well-known probabilistic CD models. Besides this, the authors of the Commentary argue that the (dichotomous) BLIM is equivalent to a DINA model if the items are regarded as binary single-attribute items, each with a distinct attribute. Among other things, this rejoinder, by drawing upon Heller et al. (2015, 2016), provides the formal arguments showing that the BLIM is not equivalent to a DINA model, and that the PoLIM is not a paraphrase of any DINA model for polytomous items. The best perspective under which such models should be considered is that of “marginal models” in the sense specified by Gu and Xu (2020).

## 2 Main concepts in knowledge structure theory

In knowledge structure theory (KST, Doignon & Falmagne, 1999, 2012), the knowledge structure is a pair 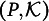 where *P* is a set named the *domain of knowledge*, and 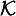 is a family of subsets of *P* that contains, at least, the empty set 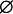 and the domain *P*. Each subset 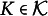 is named a *knowledge state*. In concrete applications of the theory, the set *P* is regarded to be the collection of all the problems, questions, quizzes that can be formulated in a given area of knowledge (e.g., geometry). Then, the knowledge state of an individual is the subset 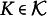 of problems in *P* that the individual masters. It can be regarded as the possibly multidimensional “ability” of the individual.

It should be observed that, in general, the knowledge structure 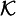 does not contain all possible subsets of *P*. That is, in concrete applications, 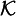 is a strict subset of the entire power set on the set *P*. The method and criteria that are used for deciding whether the subset 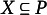 is a knowledge state or not can be either theoretical or data-driven, depending on the purpose of the application. The most elementary theoretical method consists in the specification of the so-called “surmise relation”, a quasi-order (reflexive and transitive) relation 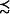 of the set *P* of problems whose interpretation is as follows: given any two problems 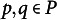 , 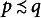 if (excluding random error) failing *p* entails failing *P*.

Example 2.1. The following example is identical to that provided by Chiu et al. (2023). For 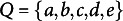 , consider the surmise relation 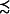 defined as follows: 

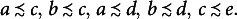

Notice that relations like 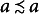 and 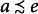 , that can be inferred by transitivity and or reflexivity are omitted. Not all subsets of *P* are “consistent” with this relation. A subset 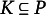 is consistent with 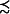 if for every problem 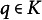 , all the predecessors of *P* are also in *K*, namely, the following implication must hold true for all pairs 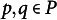 of problems: 

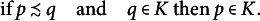

**Figure 1:**
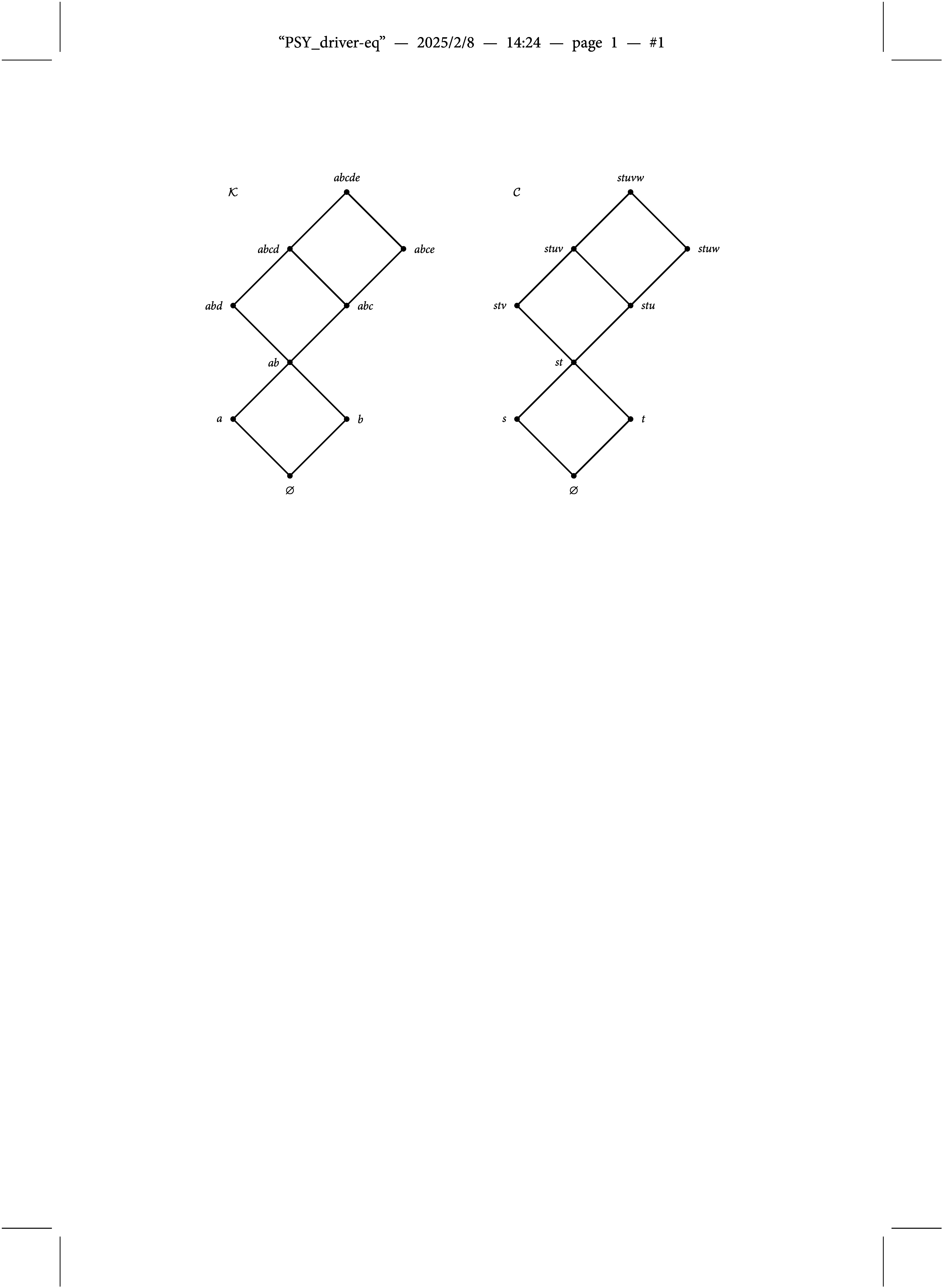
Knowledge structure 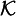 on the set of items 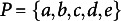 and isomorphic competence structure 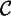 on the set of skills/attributes 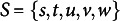 .

Of the 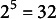 subsets of *P*, the only ones that are consistent with 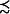 are the following nine: 

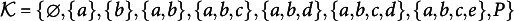

that form the knowledge structure 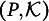 derived from 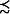 (see Figure 1 for an illustration). This knowledge structure has two fundamental properties: the intersection of whatever subset 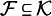 of knowledge states is itself a knowledge state (closure under intersection). Similarly, the union of 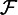 is itself a knowledge state (closure under union). Knowledge structures of this type are named *quasi-ordinal knowledge spaces*. The relationship between the surmise relations of *P* and the quasi-ordinal knowledge spaces on *P* is one-to-one (Birkhoff, 1937; Doignon & Falmagne, 1999).

## 3 How KST and CD models are interlocked?

There is a close connection between KST and CD models, which has been spelled out and developed formally by Heller et al. (2015) for the case of two particular probabilistic models in the two theories: The so-called a competence-based extension of the BLIM (CBLIM) for KST, and the multiple strategy DINA model (de la Torre & Douglas, 2008) for CD models. It was shown that the two models at hand are formally equivalent. They are essentially the very same model, expressed by using different notations and different terminologies. In all probability, prior difficulties in recognizing such equivalence just laid in the substantially different formal and notational approaches followed by the two theories.

The present section summarizes the main theoretical results of Heller et al. (2015). The fundamental concept upon which a connection between the two theories has been built is rather simple. KST is mostly a set-theoretical theory. Its deterministic skeleton is based on assumptions, definitions, and results that largely draw upon set, order, and lattice theory. There is a reason for this: KST originated in an area of mathematical psychology that grew out of measurement theory as it was conceived, for instance, in the Foundations of Measurement (Krantz et al., 1971). In that particular area, the focus was especially on qualitative structures and on how to measure them through numbers. The whole machinery was set theoretically oriented.

On the other side, CD models were mostly developed under the item response theory (IRT) framework, which is inherently probabilistic and numerical. Numerical structures like vectors and matrices are the most common tools of the various models and methods developed within item response theory. The most relevant numerical structures that are of some interest here are the binary vectors and the binary matrices, not only because they populate a wide range of CD models that are around, but also because they are easily related to deterministic KST concepts.

Thus, at the ground of the connection between the two approaches there is the elementary observation that binary vectors of a given length *n* can be put in a bijective correspondence with subsets of a set 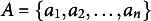 . The bijection can be easily obtained by constructing, for each subset 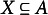 , its *indicator vector*, that is a binary vector 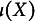 with one component for each element in *A*, where this component is one if the corresponding element of *A* is in *X*, and 0 if it is not. This simple observation makes it easy to switch from the primitive concepts of one theory to those of the other theory. This exercise was already carried out by Heller et al. (2015), who established some fundamental correspondences. Let 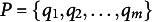 be a set of items, and 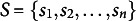 be a set of skills.

- The *observed response pattern* is a binary vector 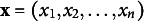 in CD models, and it is a subset 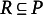 in KST. In both cases, it represents the (dichotomous true/false) responses of an individual to a set *P* of *m* items. In particular, 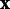 and *R* represent the same response pattern if 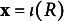 . Considering Example 2.1 on the domain 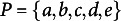 of five items, the observed response pattern of an individual providing a correct answer for items *a*, *c*, and *d* is represented by the vector 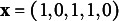 in CD models, and by the set 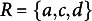 in KST.
- In CD models, the notion of an *ideal response pattern* refers to the vector 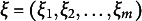 of dichomomous responses to the items that are expected if no error (e.g., in the form of guessing or slipping) occurs. In KST, this is called the *knowledge state* and it is represented as a subset 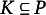 of all the items that a person is capable of solving. In Example 2.1, the knowledge state 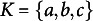 of an individual corresponds to the ideal response pattern 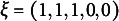 .
- The *attribute profile* is, in CD models, a binary vector 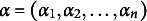 having length equal to the cardinality of the set *S* of skills. It represents the skills possessed by an individual. Let 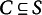 be the collection of all the skills possessed by an individual. Then in KST, *C* is called the competence state, and 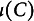 is the attribute profile. In Example 2.1, suppose that mastering all skills in the set 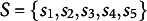 is required for solving the five items in *P*. Then, a plausible attribute profile of an individual is 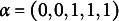 in CD models and it is 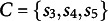 by KST.

It is worth noticing that, what in KST is called knowledge structure 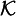 corresponds, in CD models, to the collection of all the ideal response patterns. In CD models, this collection is represented by a binary matrix, where each row represents an ideal response pattern. In Example 2.1, the collection of all the ideal response patterns corresponding to knowledge structure 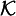 , is the binary matrix (rows are ordered like the elements in 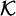 ): 

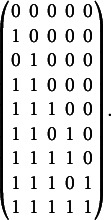



It is convenient to keep clearly distinct the level of the ideal response patterns from that of the attribute profiles. In KST, the former is referred to as the *performance level*, whereas the latter is referred to as the *competence level*. Notice that both levels represent latent constructs, which are distinguished from the observed response patterns.

In the beginning, KST was exclusively focused on the performance level of the knowledge states (ideal response patterns). From the perspective of cognitive diagnosis, ignoring the competence level of the skills may be seen as a disadvantage. If individual ability is represented by a subset of items, this representation provides no direct explanation or interpretation in terms of the psychological mechanisms underlying the response behavior. All in all, knowledge states are features of persons and as such, they should depend on skills/attributes. Since in KST the knowledge state is operationalized as a subset of items, it is item-dependent. Instead, in CD models, the notions of Q-matrix and attribute profile allow to separate individual skills from items.

Once the skill map (the Q-matrix) has been established, and its interpretation (e.g., conjunctive, rather than disjunctive) has been stated, each attribute profile delineates exactly one ideal response pattern (knowledge state). If the correspondence between attribute profiles and ideal response patterns is one-to-one, then there can be unique skill assessment (Heller et al., 2015). In this case, the attribute profile and the ideal response pattern represent exactly the very same thing, namely a multidimensional individual ability. In CD models, the multidimensional ability is further decomposed into discrete skills.

However, if the relationship is not one-to-one, then the same ideal response pattern may be associated with more than one attribute profile. In this case there cannot be unique skill assessment. It means that there is no unique way of decomposing the multidimensional individual ability into discrete skills.

To summarize, if the performance and competence levels are in a one–to–one correspondence then skill assessment is unique. Thus, the knowledge state (ideal response pattern) depends on items in as much the same way as the competence state (attribute profile) depends on items. The only situation where the performance level of the ideal response pattern and the competence level of the attribute profile do not correspond with one another is when skill assessment is not unique. Here however the problem is located at the competence level, where we have more than a single interpretation (attribute profile) for the same ideal response pattern. At the performance level, the representation remains unique.

## 4 The BLIM and the DINA model

Chiu et al. (2023) claim that the BLIM is equivalent to a DINA model, to which certain modifications and restrictions apply. More precisely, their main statement is that the

“BLIM is […] equivalent to the DINA model when the BLIM-items are conceived as binary single-attribute items, each with a distinct attribute” (p. 656).

However, regarding the BLIM as DINA model with single-attribute items is misleading, because the BLIM makes no assumptions about underlying attributes whatsoever. The very same issue arises when considering the PoLIM as a “single-attribute” version of some pre-existing polytomous DINA model. Both the BLIM and the PoLIM are totally agnostic to any psychological mechanism that might give rise to the knowledge structure they are based on. Singling out a particular link between items and attributes invites invalid conclusions.

The intimate relation between KST and CD notions in case of dichotomous items is fully characterized already by Heller et al. (2015), who prove various theorems on the existing correspondences. The BLIM is extended by introducing skills (attributes in CD terms), which are linked to the items via a so-called skill function (corresponding to a collection of Q-matrices in CD). Moreover, this approach is able to capture dependencies between skills through assuming an arbitrary competence structure, which amounts to considering an arbitrary subset of permissible attribute profiles in CD. The resulting model is called the *competence-based* BLIM (or, CBLIM).

As one of the main results of Heller et al. (2015) the CBLIM is shown to be equivalent to the multiple strategy DINA model (MS-DINA; De La Torre & Douglas, 2004). As the DINA model is a special case of the MS-DINA, it is to be conceived as a special case of the CBLIM which assumes a conjunctive rule operating on the skills/attributes. This shows that KST offers a framework for formulating models equivalent to the DINA model and special cases thereof, but these KST models cannot be identified with the BLIM.

Chiu et al. (2023) claim that “estimating BLIM using DINA requires that all BLIM-items are single-attribute items because only then 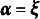 is true” (p. 658). Confining consideration to this very particular case may be fine if the aim of the paper was limited to exemplify that parameter estimation algorithms developed for the DINA model can be used for estimating the parameters of the BLIM, although the latter is equipped with its own algorithms (Heller & Wickelmaier, 2013; Stefanutti & Robusto, 2009) and publicly available routines (the R package pks; Heller & Wickelmaier, 2013). However, the cited statement as such is wrong.

Beyond the fact that the equation 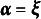 is problematic in itself (see below), the kind of constraint it implies is not necessary for aligning the predictions of a DINA model with those of a BLIM. For clarifying this issue we refer to the example of Chiu et al. (2023), which in turn was taken from Doignon and Falmagne (1999, Example 7.1). Consider the knowledge structure 

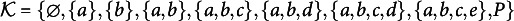

on the domain 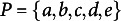 . On the left-hand side of Figure 1 the Hasse diagram of 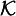 is shown, with paths of ascending line segments representing set inclusion. In this subsequent plots we use the shorthand notation 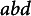 to denote the set 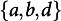 , for example.

**Figure 2:**
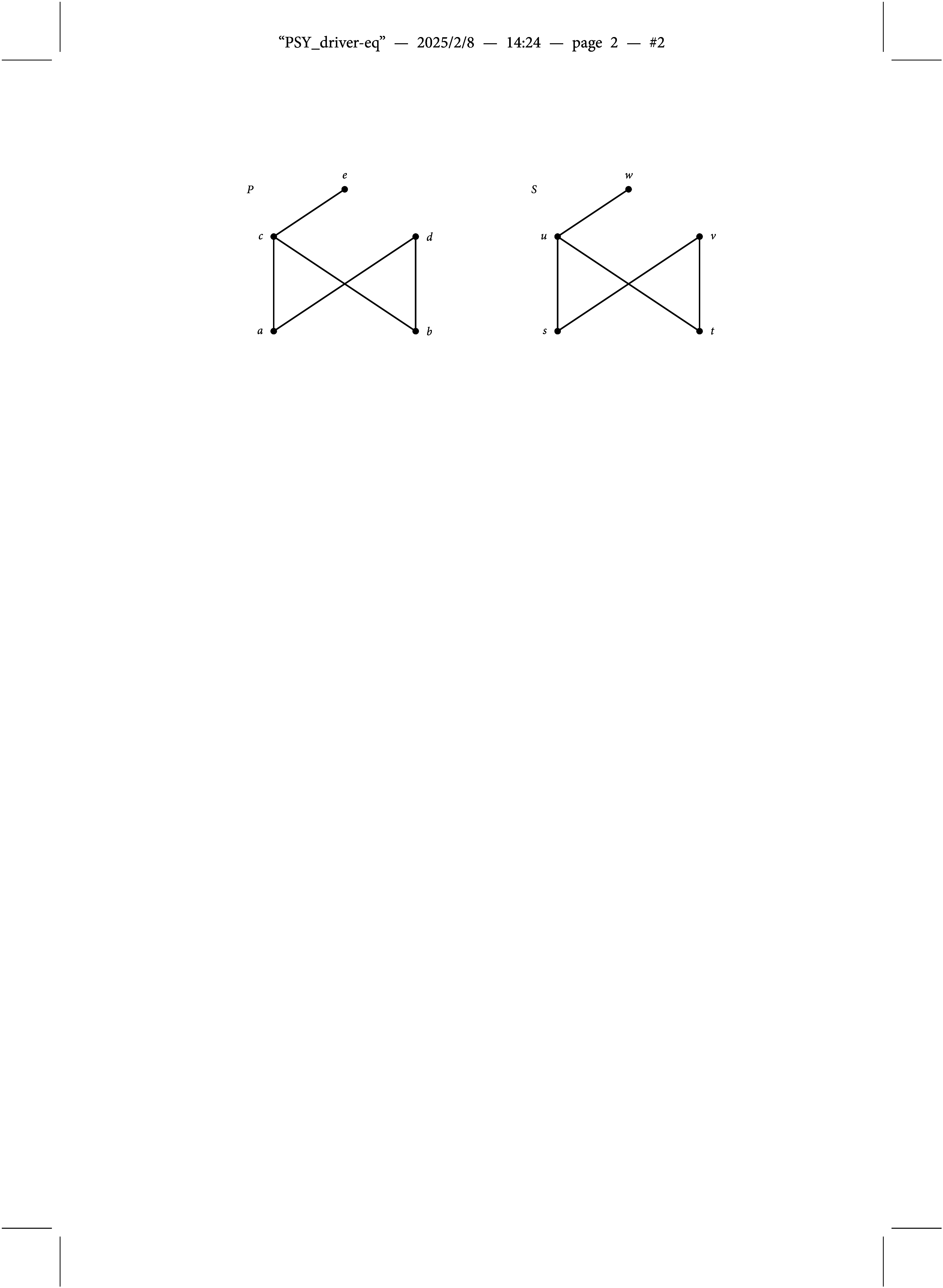
Partial orders on the set of items 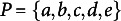 and the set of skills/attributes 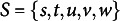 corresponding to knowledge structure 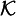 and competence structure 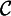 , both illustrated in Figure 1.

Notice that the knowledge structure 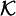 is in one-to-one correspondence to the partial order on the domain of items illustrated by the Hasse diagram on the left-hand side in Figure 2, and thus is said to be an *ordinal knowledge space* (see, e.g.,Doignon & Falmagne, 1999).

The construction of Chiu et al. (2023) then proceeds by introducing a set of skills/attributes 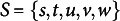 and the identity matrix 

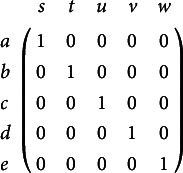

as the Q-matrix. This amounts to defining a bijection 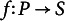 such that 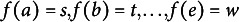 . From a structural perspective the bijection *f* establishes an isomorphism between the knowledge structure 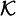 and a competence structure 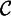 defined by the equivalence 

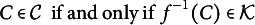

for all 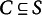 , where 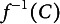 denotes the preimage of *C* under *f*. The isomorphism may be conceived as a mapping *p* from 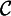 onto 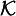 , which in KST is known as a *problem function* (e.g., Doignon & Falmagne, 1999). Comparing the Hasse diagram of the competence structure 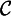 on *S* to that of the knowledge structure 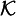 on *P* makes the isomorphism self-evident (see Figure 1). Using the notation 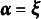 , equating attribute profiles 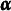 (corresponding to the competence states 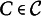 ) and ideal response patterns 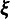 (corresponding to the knowledge states 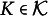 ), obscures the fact that there is no identity, but only an isomorphism. Choosing a competence structure 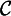 (i.e., the permissible attribute patterns) isomorphic to 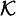 amounts to inducing a hierarchical structure on the skills/attributes, which is isomorphic to the partial order on the items corresponding to 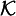 (see the Hasse diagram at the right-hand side of Figure 2). This goes unmentioned in Chiu et al. (2023), and ignoring this fact leads to a fundamental misunderstanding when they are dealing with identifiability issues (see Section 5).

**Figure 3:**
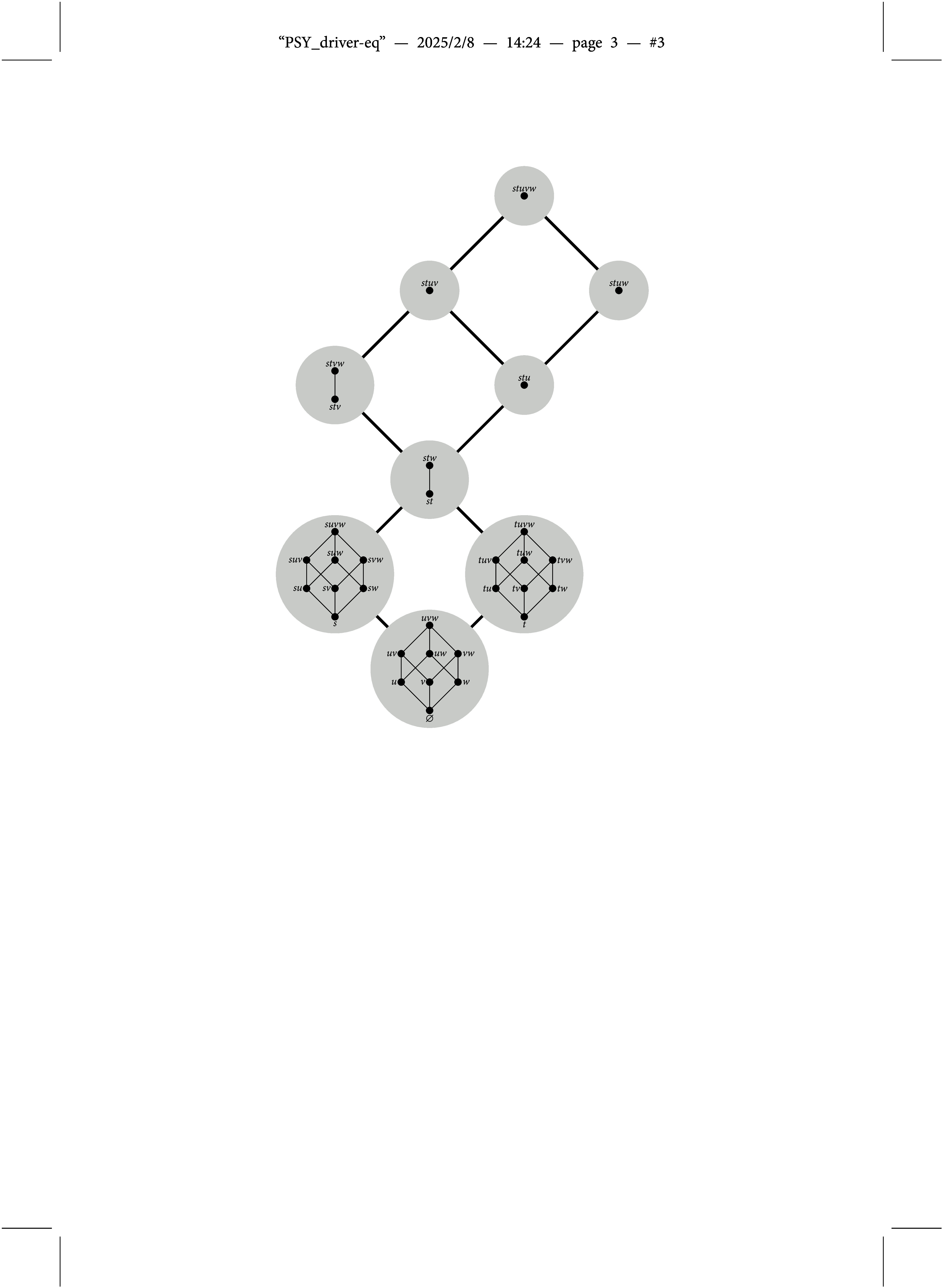
Line diagram illustrating the many-to-one relationship between competence and knowledge states (i.e., attribute profiles and ideal response patterns) for an unstructured set of skills/attributes and the Q-matrix of Equation (1).

Instead of imposing the above outlined constraints by inducing a hierarchical structure on the skills/attributes with the Q-matrix being the identity matrix, let all possible attribute profiles on the skills/attributes in *S* be permissible. Using the Q-matrix (1)

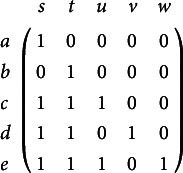

the induced ideal response patterns are the vector representations of exactly the knowledge states in 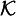 (Heller, 2022, Corollary 2). Notice that this Q-matrix is a *densified* version of the identity matrix in the sense of Gu and Xu (2020), and plays a prominent role in the so-called *restricted Q-matrix design* in the context of *attribute hierarchy models* (Leighton et al., 2004). This example makes clear that the very same knowledge structure can arise from substantially different assumptions on the underlying skills/attributes: while Chiu et al. (2023) assume single-attribute items, the Q-matrix of Equation (1) assumes multiple-attribute items. The equivalence of the two DINA models with respect to the ideal response patterns is due to the fact that the knowledge structure 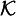 is closed under intersection, a property which results as a necessary consequence of applying a conjunctive rule to an unstructured set of skills/attributes (Gediga & Düntsch, 2002; Ünlü et al., 2013). Notice that in this case the problem function *p* from 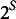 to 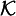 mapping competence states to knowledge states (i.e., attribute profiles to ideal response patterns) is not one-to-one, but many-to-one as illustrated in Figure 3. Gray circles are in one-to-one correspondence to the knowledge states in 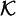 shown in Figure 1, and all the competence states plotted in each of them form an equivalence class, as all of them are mapped to the respective knowledge state. For example, all the competence states included in 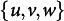 are mapped to the empty knowledge state 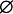 (i.e., 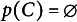 for all 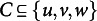 ). This means that the hierarchical structure of the skills/attributes can be represented in either the set of permissible attribute profiles (as in Figure 1), or in the Q-matrix (as in the densified version), or in both. In fact, given the Q-matrix of Equation (1), there are many more competence structures that induce the very same knowledge structure 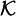 . Take for example all the competence structures on *S* that include the structure 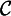 of Figure 1. Notice that the latter results from selecting the minimal competence states of each of the equivalence classes in Figure 3 (i.e., from each of the gray circles). All the instantiations of the DINA model built on these competence structures may be considered equivalent: not only because they induce the same knowledge structure, but also concerning the probabilistic framework defined on top of it. This is illustrated in the sequel.

Let 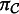 denote the probability distribution over the competence states in 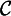 , 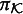 the probability distribution over the knowledge states in 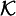 , and 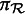 the probability distribution over the response patterns in 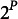 . With 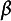 and 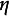 the vectors of careless error and lucky guess probabilities (collecting all the 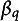 and 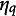 , 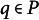 ), the CBLIM is characterized by the parameters 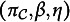 , and the BLIM induced by the CBLIM (via 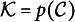 ) is characterized by the parameters 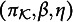 . In general the relation between these two parameter spaces is given by the equation (2)

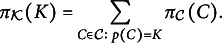

That is, we have the composition of the mappings (3)

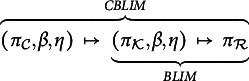

In the situation illustrated in Figures 1 and 3 this means that the probability of the knowledge state 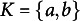 , for example, is given by 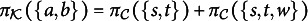 .

While in any case the BLIM careless error and lucky guess parameters are identical to the DINA slipping and guessing parameters, the BLIM probability of knowledge state *K* in 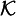 is obtained by summing over the DINA probabilities of all attribute profiles 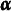 that are mapped to the ideal response pattern 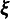 corresponding to the knowledge state *K* (Heller, 2022; Heller et al., 2015). This allows for fully recovering the BLIM parameter estimates, also in cases where the very same BLIM model is induced by distinct DINA models, which may be based on substantially different assumptions as outlined above.

Tables 1 and 2 show estimates of item parameters and probabilities of knowledge states and ideal response patterns, respectively, when replicating estimation for the example assuming a uniform initial distribution on all possible attribute profiles in the DINA model and the densified Q-matrix of Equation (1). The corresponding initial distribution on the knowledge states in the BLIM is derived using Equation (2). Otherwise, the same setup as described in Chiu et al. (2023) is used. Results again show that all the estimates of the BLIM and DINA parameters are essentially identical, although distinct from those listed in Chiu et al. (2023). The reason for the latter finding is discussed in Section 5.

**Table 1:** Item parameter estimates of the BLIM and the DINA model, with the Q-matrix of Equation (1) and all attribute profiles being permissible

	BLIM			DINA model	
	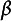	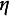		*s*	*g*
a	.1649	.1787		.1648	.1787
b	.1631	.1688		.1631	.1688
c	.1888	.0000		.1888	.0001
d	.0704	.0000		.0704	.0001
e	.0881	.0199		.0881	.0199

**Table 2:** Results of estimating probabilities of the knowledge states via the BLIM and of the ideal response patterns via the DINA model, with the Q-matrix of Equation (1) and all attribute profiles being permissible

BLIM			DINA model	
*K*	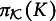		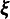	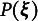
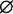	.0761		00000	.0761
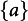	.1004		10000	.1004
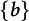	.0947		01000	.0948
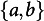	.0480		11000	.0480
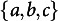	.1352		11100	.1352
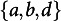	.1137		11010	.1137
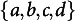	.1336		11110	.1335
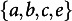	.1432		11101	.1432
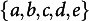	.1551		11111	.1551

The coinciding parameter estimates demonstrate that the claim of Chiu et al. (2023), that for mimicking the BLIM with the DINA model, all items need to be conceived as single-attribute items, is wrong. In fact, we have shown above that there may be a variety of distinct DINA models that can mimic a given BLIM. The general mechanism for setting them up is to mirror the information captured by the BLIM’s knowledge structure at the skill/attribute level by coding it by either the set of permissible attribute patterns, or the Q-matrix, or even both. Doubling up the given behavioral information, however, cannot generate new insights. So, any interpretation of the Q-matrix (such as being composed of single- or multi-attribute items, for example) may be meaningless.

The BLIM does not need to come with the specification of any theoretical cognitive assumptions. As a marginal model, which is in line with a vast variety of cognitive theories, it is characterized by a high degree of flexibility. This allows for closely mirroring the structure underlying the observed responses. A knowledge state identifies the items that are mastered or not mastered by an individual, and a knowledge structure tells you whether the mastery of certain items implies the mastery of others. The knowledge structure underlying a BLIM may be established in a completely data-driven way, without the need of resorting to theoretical assumptions on the cognitive processing (e.g., Falmagne et al., 2013, Section 9.3). This results in knowledge structures of high empirical validity and provides a basis for efficient and precise knowledge assessment as well as for personalized learning. This is demonstrated by highly successful large-scale applications (Falmagne et al., 2013; Falmagne & Doignon, 2011, like the ALEKS^1^ system.

Of course, in a second step one may be interested in studying the cognitive mechanisms that bring about the knowledge structure, but this may introduce uncertainties. For example, if considering a DINA model with all attribute profiles being permissible and the Q-matrix of Equation (1) then, in case that more than one attribute profile is mapped to the same ideal response pattern (collections in each of the gray circles in Figure 3), the respective probabilities are not identifiable. In parameter estimation of a DINA model the probabilities of the attribute profiles in these collections usually turn out to be equal, but this is nothing more than an artifact of assuming a uniform initial distribution as the default. What in principle may be identifiable are the knowledge state probabilities of the BLIM, but see Section 5 for a discussion of the current example.

In conclusion, tying the BLIM to a very special case of the DINA model as in Chiu et al. (2023) creates a misleading perspective. The BLIM is to be conceived as a marginal model for a whole class of models that involve cognitive assumptions. There is previous theoretical work that elaborates on this point but found no mention in Chiu et al. (2023). The relation of the BLIM to its competence-based extension CBLIM as well as their correspondence to CD models was characterized in full generality by Heller et al. (2015, 2016). See also Gu and Xu (2020), who introduced a conception equivalent to the BLIM in the CD context by considering what they called grouped population proportion parameters. On the basis of this concept they analyze the identifiability properties of the class of two-parameter Q-restricted latent class models (see Section 5 for further details).

## 5 Identifiability

Chiu et al. (2023) are right in observing that the BLIM is not identifiable for many types of knowledge structures. There is a broad KST literature which studies the kinds of non-identifiability that arise, and characterizes the conditions under which identifiability holds, or can be restored (see, e.g., Doignon et al., 2018; Heller, 2017; Spoto et al., 2012, 2013; Stefanutti et al., 2012, 2018). The authors refer to a similar characterization in CD by stating that “Gu and Xu (2019) showed that the DINA model is only identifiable if each attribute is used by at least three items (Condition 1 (ii); p. 471). By definition, this condition cannot be fulfilled by the DINA model with single-attribute items, as its Q-matrix is a 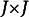 identity matrix—hence, due to the equivalence of the two models, identifiability cannot hold for BLIM either” (p. 665). A few remarks are in order here.

First, there is no basis for making inferences on the identifiability of the BLIM given that a single-attribute DINA is not identifiable. It was shown above that the two models cannot be conceived as being equivalent in any formal sense, but the BLIM may be seen as a marginal model for a whole class of CD models.

Second, the cited conclusion of Gu and Xu (2019) only holds for the case where all theoretically conceivable attribute profiles are permissible (see, e.g., Gu & Xu, 2020, p. 2089). It has been made clear above that this is not the situation considered in Chiu et al. (2023), where a hierarchical structure on the skills/attributes is imposed by partially ordering them as illustrated in Figure 2, and thus the argument is vacuous. Moreover, the presented rationale would lead to the conclusion that any BLIM is not identifiable irrespective of the underlying knowledge structure, which is obviously false.

Third, as already mentioned, there are theoretical results for hierarchies of attributes in the CD context. Gu and Xu (2020) investigate identifiability of a class of CD models (the two-parameter Q-restricted latent class models quoted above) including the DINA model for general attribute structures (i.e., arbitrary subsets of attribute profiles are permissible). They define and characterize the concept of *partial identifiability*, which corresponds to identifiability of the BLIM induced by a CBLIM (Heller, 2022). Considering the CBLIM (which includes the DINA model as a special case), it follows that the CBLIM is (locally) identifiable if and only if the composition of the mappings in Equation (3) is injective (Heller et al., 2015, Corollary 1). It was shown that this holds if and only if the induced BLIM is identifiable and the problem function *p* is injective. Since *p* as an isomorphism is injective by the construction employed by Chiu et al. (2023), the CBLIM is (locally) identifiable if and only if the induced BLIM is (locally) identifiable. Recasting this in CD terms, the question actually is whether the DINA model is partially identifiable, or not.

The KST literature provides results showing that the BLIM defined on the considered knowledge structure 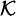 of Example 2.1 is not identifiable, both locally and globally (Heller, 2017; Spoto et al., 2012). The following notions are central to characterizing this case of non-identifiability. A knowledge structure 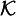 is said to be *backward-graded* in an item 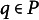 if for each state 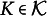 the subset 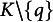 is also a state in 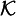 , and it is said to be *forward-graded* in an item *P* if for each state 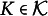 the subset 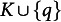 is also a state in 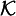 (Spoto et al., 2012). The items in which 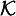 is backward- or forward-graded are easily identified by inspecting the corresponding partial order on *P* illustrated in Figure 2. Heller (2017, Corollary 1) shows that the ordinal knowledge space 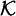 is backward-graded in the maximal elements (i.e., in items *d* and *e*), and it is forward-graded in the minimal elements (i.e., in items *a* and *b*) of the corresponding partial order. Thus, the BLIM in the empirical demonstration of Chiu et al. (2023) indeed is not identifiable, and thus different initial parameter values in the estimation procedure of both the BLIM and the DINA model may lead to different final estimates. In particular, theoretical results of Spoto et al. (2012) and Heller (2017) predict that the affected item parameters are those of the items in which the knowledge structure 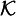 is backward- or forward-graded. Comparing Table 1 with the respective table in Chiu et al. (2023, p. 659) confirms this prediction. Discrepancies occur for the careless error and slipping probabilities (columns 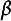 and *s*) of the maximal items *d* and *e* (previously 0.0798 and 0.0886), and for the guessing probabilities (columns 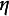 and *g*) of the minimal items *a* and *b* (previously 0.1031 and 0.0951). These discrepancies are due to the differences in the initial knowledge state probabilities induced on the BLIM by the different instantiations of the DINA model. While in Chiu et al. (2023) the initial distribution is uniform, it results from Equation (2) via the assumption of a uniform distribution on all possible competence states (i.e., all permissible attribute profiles) in the above considered alternative model. For example, for the empty knowledge state 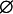 the initial probability equals 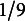 in the first case, and 
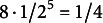
 in the second case. The obvious discrepancies of the estimated knowledge state probabilities in Table 2 from those listed in Chiu et al. (2023, p. 659) are explained by the parameter trade-offs theoretically characterized by Spoto et al. (2012, Theorems 1 and 3) and Heller (2017, Propositions 1 and 3) for global and local non-identifiability, respectively.

Pointing to a wealth of results available in both the KST and the CD literature, the discussion in the present section lets the concluding remark of Chiu et al. (2023) that “which models are equivalent when attributes have a hierarchy, and which Q-matrices lead to identical results is currently uncharted territory” (p. 665) appear rather strange. In fact, their application of the DINA model assumes an attribute hierarchy as illustrated in Figure 2, and theoretical results for this and even more general cases are readily available (Gu & Xu, 2020; Heller, 2022; Heller et al., 2015).

## 6 PoLIM and the polytomous DINA model

An extension of the BLIM to items with more than two response alternatives was proposed by Stefanutti, de Chiusole, et al. (2020). The provided model, named the PoLIM (politomous local independence model), is derived from assumptions that are minimally sufficient for extending the BLIM to polytomous items. The PoLIM can be applied to a very large variety of response formats. For instance, the item responses can be totally ordered, partially ordered or not ordered at all. Different items may have different numbers of response alternatives. There could be a mixture of dichotomous and polytomous items. No skills are assumed and no Q-matrices need be specified, although the model can be extended to either dichotomous or polytomous skills.

In this connection, a polytomous version of the DINA model was proposed by Chiu et al. (2023) in a commentary to Stefanutti, de Chiusole, et al. (2020). Moreover, there exist many varieties of CD models for accommodating polytomous items, that were developed before the PoLIM (see, e.g., (Chen & de la Torre, 2018; Chen & Zhou, 2017; von Davier, 2008)).

Chiu et al. (2023) try to generalize their base argument that the BLIM is a DINA model with single-attribute items to the polytomous case (i.e., to the PoLIM). In constructing a counterexample to their argument, like in the dichotomous case we delineate the same polytomous structure obtained by Chiu et al. (2023) with a different Q-matrix and a different set of attribute profiles. The aim of the example is to show that there are at least two different Q-matrices (the one in the example below and the one by Chiu et al. (2023)) that delineate exactly the same polytomous structure (set of polytomous ideal response patterns) at the performance level. There is no “standard” way of defining a Q-matrix, when the item responses are polytomous. The approach followed here relies upon recent developments described in Stefanutti et al. (2023), but differs to some extent from the one described in the Commentary.

Chiu et al. (2023) considered a set 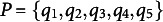 of five items and a set 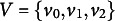 of linearly ordered response values. The first four items in the example were dichotomous, whereas the last one was trichotomous. Here, we consider the Q-matrix displayed in Table 3, which assigns a subset of 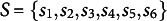 to each of the admissible item-response pairs for *P* and *V* listed in the first column.

**Table 3:** Q-matrix delineating the same set of ideal response patterns as in the example by Chiu et al. 2023

Item-response pair	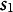	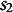	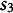	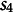	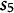	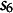
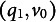	0	0	0	0	0	0
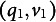	1	0	0	0	0	0
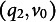	0	0	0	0	0	0
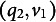	0	1	0	0	0	0
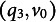	0	0	0	0	0	0
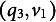	1	1	1	0	0	0
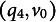	0	0	0	0	0	0
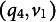	1	1	0	1	0	0
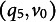	0	0	0	0	0	0
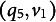	1	1	1	0	1	0
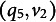	1	1	1	0	1	1

It is worth noticing that, in the example by Chiu et al. (2023), the number of skills was five whereas in this example it is six. Moreover, in the example by Chiu et al. (2023), the Q-matrix can take on any value in *V*, whereas in the present counterexample it is dichotomous. Nonetheless, it produces polytomous ideal response patterns.

The full power set on the six skills is considered as the set of permissible attribute profiles, containing a total of 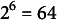 attribute profiles. This choice is clearly different from the one in the example by Chiu and colleagues, which contains 11 attribute profiles in the whole. In the sequel, the notation 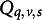 refers to the value of the Q-matrix for item *q*, response *v*, and attribute *s*. Thus, for instance, 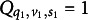 , 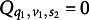 , 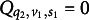 , etc. Define then the function 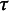 such that, for any item *P*, any response value 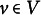 , and any attribute 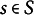 , it holds that 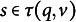 if and only if 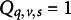 . Then, we first need to determine what the “ideal response” to item *P* will be when the attribute profile is 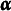 , given that the set *V* contains more than two alternative answers. Such an ideal response is here defined as 

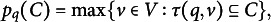

where 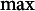 is the usual “maximum” function, and 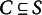 is the set representation of the binary vector 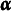 (i.e., 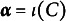 ). Thus, 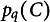 provides *P* with the maximum response value among all those response values that could be provided by an individual whose attribute profile is 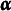 . The set representation (knowledge state) of the ideal response pattern “delineated” by 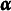 is then obtained as 

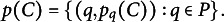

One should be careful with these definitions because, in general, 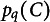 need not exist. The existence is only guaranteed if there is a maximum in the set 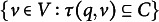 . In our running example, however, this always holds true because the three response values in *V* are linearly ordered, and the minimum value is such that 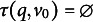 for all items *P* (more general cases are considered in Stefanutti et al. (2023)). To show how all of this applies concretely, suppose that the attribute profile is 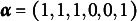 . Its set representation is 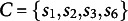 . Then we have 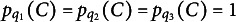 , 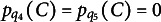 . This yields 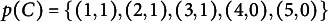 , whose corresponding vector representation is 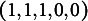 . In the whole, by applying this method to each of the 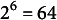 attribute profiles considered in this example, the obtained polytomous structure turns out to be identical to the one in the example provided by Chiu et al., that is: 

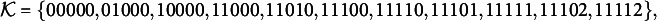

whose elements are shorthand vector representations of the polytomous states. Like in the dichotomous example, there is an alternative skill interpretation of the PoLIM. In such interpretation there are items requiring more than a single skill, showing that the PoLIM is not a polytomous DINA model where each item requires a single skill.

As stated above, depending on how the elements in *V* are ordered, the mapping 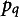 may be undefined for some attribute profiles. Suppose in fact that *V* is unordered (like with categorical or nominal responses). Then, for sure, 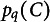 will be undefined because the set 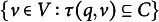 has not a maximum element. This fact does not affect the PoLIM, which remains applicable also with unordered and, more generally, with partially ordered response categories. Unfortunately, the same conclusion cannot be drawn with respect to the polytomous DINA model presented by Chiu and colleagues in the Appendix of their Commentary.

In Section 4.2., Chiu et al. (2023) claim that “… here, the general case is concerned that also includes non-ordered response categories; hence, the indices 
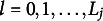
 should be merely interpreted as category labels”. However, if such an interpretation is applied, then the polytomous DINA model may provide meaningless ideal response patterns. The reason is in the way ideal response patterns are obtained from the attribute profiles. The equation that formally relates the former to the latter can be found in the Appendix of the cited article and it is repeated here for convenience (with the same notation used in the commentary): (4)

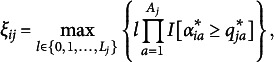

where 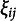 is the *j*th entry in the ideal response pattern of individual *i*, 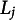 is the highest level of the polytomous ideal response, 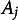 is the total number of attributes for item *j*, 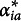 and 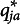 are, respectively, the rearranged attribute profile for individual *i*, and attribute *a*, and the rearranged polytomous Q-matrix entry for item *j* and attribute *a*.

As an example, suppose that in a study on preference, each item in a questionnaire asks the respondent to choose one element out of a set of three. There is no a-priori order on the three response alternatives, because it may vary from one individual to another according to preference. Clearly, in such a situation, the numbers in the set 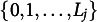 that appears as the subscript of the maximum in Equation (4) can only have nominal value (e.g., although one is less than two, the response alternative associated with the number one and that associated with the number two cannot be ordered in a unique way). In other words, with unordered response alternatives, although a maximum always exists in the finite set 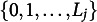 , it does not exist among the unordered response categories that are represented by those numbers.

Suppose then that 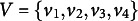 is a set of four response categories (notice that *V* is a non-numerical set), and denote by 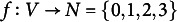 the mapping that assigns a number to each of the categories in *V*. Given the unordered nature of the elements in *V*, the mapping *f* can be any bijection. The numbers only serve as labels. Therefore, if 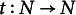 is a bijection, then the function composition 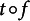 is still a bijection between *V* and *N*, meaning that *f* and 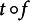 are equally good in representing *V*. If, like in our example, the choice of the numbers representing the elements in *V* is restricted to the set *N*, then 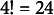 distinct bijections are possible. It is questioned whether the response category represented by 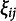 is invariant under bijective transformations 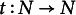 . We show that, unfortunately, the answer is negative.

**Table 4:** A polytomous Q-matrix for a set of four items and three polytomous skills

Item	Response	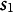	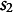	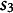
*a*	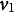	2	0	0
*a*	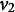	0	1	0
*a*	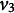	0	0	2
*b*	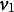	2	0	0
*b*	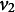	0	1	0
*b*	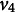	1	0	1
*c*	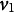	2	0	0
*c*	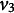	0	0	2
*c*	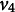	1	0	1
*d*	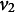	0	1	0
*d*	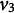	0	0	2
*d*	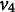	1	0	1

*Note*: There are three response categories for each item, taken from the unordered set 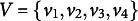 .

Suppose that the questionnaire contains four polytomous items 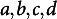 , each of which presents the participant with three of the four options in the set *V* (e.g., one of the items could present the participant with the options 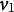 , 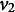 and 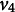 ). The response of the participant consists of choosing one of the three options. Suppose furthermore that three polytomous attributes 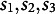 of individuals are associated with the responses to the four items. Each of the three attributes can take on a value in the set 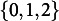 . A polytomous Q-matrix that associates attributes to item-response pairs is displayed in Table 4. So, for instance, the Q-matrix predicts that, for responding 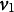 to item *a*, an individual’s attribute profile must score at least two in attribute 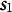 . For responding 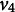 to item *b* the individual’s profile must score at least one in both attributes 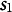 and 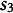 , and so on.

With 3 trichotomous attributes, the total number of attribute profiles turns out to be 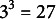 . Equation (4) can now be applied to each of the 27 attribute profiles for obtaining an ideal response pattern, by applying the Q-matrix specified in Table 4. This, however can only be done after having chosen one of the 24 alternative bijective correspondences between the options in *V* and the numbers in *N*. Assume that the mapping *f* such that 

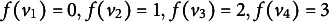

is applied. In this case, the attribute profile 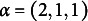 delineates the ideal response pattern 
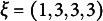
 . The details of the derivation are only described for item *a*. We have 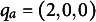 , and hence 
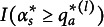
 equals 1 for 
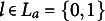
 and it equals 0 for 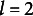 . By taking the maximum (between 0 and 1), one obtains 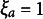 . For the remaining three items a similar procedure is applied. At this point we can map the ideal response pattern 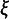 back to the original options in *V* through the inverse bijection 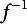 . We obtain 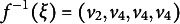 . The conclusion is: Ideally, an individual with attribute profile 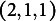 will choose 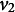 in item one, and 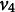 in all other items.

**Table 5:** List of all the ideal response patterns 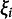 , in terms of the non-numerical values in set *V*, that are obtainable through the application of the mappings *f* (columns 1 to 4) and *g* (columns 5 to 8)

*f*					*g*			
*a*	*b*	*c*	*d*		*a*	*b*	*c*	*d*
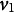	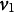	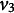	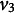		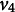	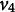	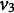	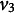
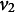	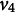	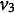	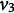		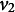	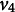	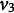	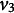
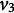	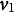	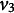	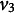		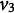	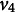	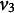	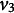
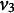	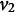	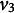	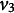		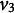	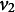	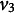	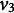
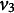	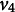	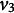	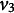		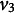	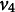	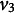	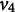
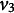	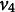	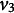	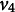		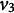	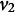	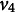	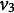
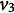	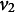	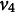	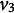		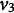	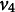	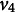	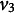
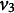	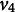	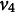	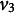		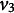	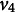	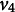	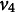
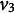	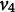	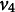	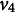					

*Note*: The two mappings generate totally different sets of ideal response patterns.

Suppose now that the mapping *g* such that 

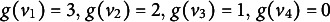

is applied, rather than *f*. Of course between the two there exists a bijection. In this alternative case, by keeping every other aspect identical, the attribute profile 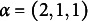 delineates the ideal response pattern 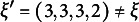 . This different ideal response pattern is mapped back to 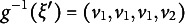 . A totally different set of choices, compared to 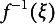 . However, the only difference between the two cases is in the choice of the bijective mapping from options in *V* to numbers in *N*. For the sake of completeness, Table 5 lists the complete sets of ideal response patterns that are obtained with bijections *f* (columns 1 to 4) and *g* (columns 5 to 8). As it can be seen, totally different ideal response patterns are obtained. The two sets do not even match in size. Concluding, contrary to what Chiu et al. (2023) claim, the PoLIM is not a paraphrase of a DINA model in cognitive diagnosis for polytomous items. The former cannot be obtained from the latter, in general. This fact becomes particularly evident when the response categories do not form a totally ordered set. To conclude, like in the BLIM case, the PoLIM is better understood as a marginal model for an entire class of CD models.

## 7 Conclusions

Chiu et al. (2023) comment on the relationship between probabilistic KST models, such as the BLIM and the PoLIM, and CD models. Their commentary, however, ignores well-established facts and invites misunderstandings. Moreover, it fails to cite relevant literature, including Heller et al. (2015, 2016), who formally established the equivalence between a CBLIM and the MS-DINA model.

This rejoinder has focused on three critical aspects covered by the Commentary: (1) the relationship between the BLIM and the DINA model; (2) the identifiability of the BLIM; (3) the relationship between the PoLIM and the polytomous DINA model proposed in the Commentary.

Concerning (1), the fact that the BLIM is agnostic with respect to skills already shows that conceiving the BLIM as a DINA model with single-attribute items is misleading at least. Putting aside problematic statements and interpretations, the contribution of Chiu et al. (2023) merely demonstrates how to set up the DINA to mimic the BLIM. However, the suggested setup need not even be unique. Section 4 showed that there may be a variety of DINA models that allow for estimating the BLIM parameters (see also Heller, 2022). Actually, there is an even larger class of CD models that share this property, because the BLIM may be conceived a marginal model for the so-called two-parameter Q-restricted latent class models (Gu & Xu, 2020).

As for (2), Chiu et al. (2023) conclude that in general identifiability cannot hold for the BLIM. Again, previous literature on the BLIM’s identifiability, but also on that of the DINA model in case of attribute hierarchies, was ignored here. It is well-established that the identifiability of the BLIM strictly depends on the underlying knowledge structure, including many cases where it is indeed identifiable. Section 5 explains in detail why the corresponding line of reasoning in Chiu et al. (2023) is flawed.

Finally let us turn to the relationship between the PoLIM and the polytomous DINA model in the sense of Chiu et al. (2023). Contrary to what was claimed, the latter seems to be a new model that was created ad-hoc by these authors. This means that this DINA model for polytomous items did not exist at the time when the PoLIM was introduced by Stefanutti, Anselmi, et al. (2020). So, rather than considering the PoLIM as “simply a paraphrase of a DINA model in cognitive diagnosis for polytomous items” (Chiu et al., 2023, p. 656), the question actually is whether it is the other way round. However, the polytomous DINA model suggested by Chiu et al. (2023) is only applicable if response categories are totally ordered, a restriction that does not apply to the PoLIM. Thus there are empirical situations (e.g., in case of nominal response categories) where the PoLIM is applicable, while the polytomous DINA model of Chiu et al. (2023) is not, and neither model is a “paraphrase” of the other. Moreover, in the same way as the BLIM is a marginal model for a whole class of CD models, the PoLIM may be conceived as a marginal model for certain polytomous CD models.

To conclude, the above discussion shows that intensifying the communication between the two camps can be useful for unifying and generalizing the approaches of both KST and CD.

## Footnotes

1 Acronym for Assessment and LEarning in Knowledge Spaces, www.aleks.com.
